# Supplementary material for: Is There a Relationship between Psychotic Disorders and the Radicalization Process? A Systematic Review
Source: Medicina (Kaunas). 2024 Jun 1;60(6):926. doi: 10.3390/medicina60060926 (PMC11205664; doi:10.3390/medicina60060926)
Supplement: Supplementary file 1 [file medicina-60-00926-s001.zip › medicina-2990505-supplementary.pdf]

Table S1. Risk of bias assessments in observational studies

Author, Year of publication *(Type of Study)*	**Risk of Bias assessed using ROBINS-E tool**							
	Bias due to confounding	***Bias arising from measurement of the exposure***	***Bias in selection of participants***	***Bias due to post-exposure interventions***	***Bias due to missing data***	***Bias arising from measurement of the outcome***	***Bias in selection of the reported result***	**Overall Risk of Bias**
**Bronsard G., 2022** ***(Case-control Study)***	Some concerns	Low	Low	Low	Low	Low	Low	Low risk of bias except for concerns about uncontrolled confounding
**Cerfolio N., 2022** ***(Retrospective Observational study)***	Some concerns	Low	High	Low	Some concerns	Low	Low	High
**Garcet S., 2021** ***(Prospective Observational study)***	Some concerns	Low	Some Concerns	Low	Low	Low	Low	Some concerns

**Glick I., 2021** *(Retrospective Observational study)*	Some concerns	Low	High	Low	Some concerns	Low	Low	High
**Morris A., 2020** *(Cross-sectional Observational study)*	Some concerns	Low	High	Low	Some Concerns	Some concerns	Some concerns	High
